# Single incision laparoscopic surgery using conventional laparoscopic instruments versus two-port laparoscopic surgery for adnexal lesions

**DOI:** 10.1038/s41598-021-82204-5

**Published:** 2021-02-18

**Authors:** Kuan-Ju Huang, Kuan-Ting Lin, Chin-Jui Wu, Ying-Xuan Li, Wen-Chun Chang, Bor-Ching Sheu

**Affiliations:** 1 Department of Obstetrics and Gynecology, National Taiwan University Hospital, National Taiwan University College of Medicine, 7 Chung-Shan South Road, Taipei, Taiwan; 2 National Taiwan University Hospital Hsin-Chu Branch, Hsichu City, Taiwan

**Keywords:** Reproductive disorders, Surgery

## Abstract

Single incision laparoscopic surgery (SILS) has emerged as least invasive interventions for gynecologic disease. However, SILS is slow to gain in popularity due to difficulties in triangulation and instrument crowding. Besides, the costly instruments may influence patients’ will to have this procedure, and limit other medical expense as well. To optimize outcome and reduce cost, the objective of this study is to evaluate the feasibility and safety for patients undergoing adnexal surgeries using conventional laparoscopic instruments with SILS (SILS-C), and to compare with those of patients subject to TP using conventional laparoscopic instruments (TP-C). This is a retrospective case–control study. The data dated from April 2011 to April 2018. Patients who received concomitant multiple surgeries, were diagnosed with suspected advanced stage ovarian malignancy, or required frozen sections for intraoperative pathologic diagnosis were excluded. Demographic data, including the age, body weight, height, previous abdominal surgery were obtained. The surgical outcomes were compared using conventional statistical methods. 259 patients received SILS-C. The operating time was 63.83 ± 25.31 min. Blood loss was 2.38 ± 6.09 c.c. 58 patients (24.38%) needed addition of port to complete surgery. 384 patients received TP-C. Compared with SILS-C, the operating time was shorter (57.32 ± 26.38 min, OR = 0.984, CI = 0.975–0.992). The patients were further divided into unilateral or bilateral adnexectomy, and unilateral or bilateral cystectomy. Other than the operating time in unilateral cystectomy (66.12 ± 19.5 vs. 58.27 ± 23.92 min, *p* = .002), no statistical differences were observed in the subgroup analysis. Single incision laparoscopic surgery using conventional laparoscopic instruments is feasible and safe as initial approach to adnexal lesions. In complex setting as unilateral cystectomy or pelvic adhesions, two-port access may be considered.

## Introduction

Laparoscopic surgery is one of the greatest advancements in contemporary surgery; providing the advantages of a shorter recovery time, less postoperative pain and fewer complications than conventional laparotomy. Single incision laparoscopic surgery (SILS) has emerged as one of the most common and least invasive interventions for gynecologic disease^1^. Compared with conventional laparoscopic surgery (CL), SILS reduces postoperative pain, requires shorter hospital stays, and achieves a better cosmetic outcome^2–6^. However, SILS is slow to gain in popularity due to difficulties in triangulation and instrument crowding, despite advances in laparoscopic cameras and instruments^7–9^. Besides, the published literature contains little firm evidence to convince patients of the superiority of SILS since most previous studies in the field consider only a small sample size and involve large variability in the surgical procedures and / or instruments used. Moreover, some of the studies show conflicting results in operating time, blood loss, and pain^1,3–6,10,11^. The costly instruments used in SILS may influence patients’ will to have this procedure, and limit other medical expense. To optimize outcome and reduce cost, there had been reports evaluating feasibility of SILS using conventional laparoscopic instruments other surgical fields^12–14^. Two studies addressed experience in gynecology, with few information compared to other laparoscopic platform^15,16^. The primary objective of this study is to evaluate the feasibility and safety for patients undergoing adnexal surgeries with SILS using conventional laparoscopic instruments (SILS-C). The secondary objective is to compare with those of patients subject to two-port laparoscopic surgery using conventional laparoscopic instruments (TP-C).

## Materials and methods

The study was approved by the Institutional Review Board of National Taiwan University Hospital (201812166RIND), and all methods were carried out in accordance with relevant guidelines and regulations. The study took the form of a retrospective, case–control study of patients undergoing laparoscopic surgery for adnexal disease. All enrolled patients gave their informed consent. The data used for analysis purposes dated from April 2011 to April 2018. For each patient, the decision to use SILS-C or TP-C was made according to the preference of the attending gynecologist. Patients who received concomitant multiple surgeries, were diagnosed with suspected advanced stage ovarian malignancy, or required frozen sections for intraoperative pathologic diagnosis were excluded. Demographic data, including the age, body weight, height, body mass index, parity, history of previous abdominal surgery, type of surgery, uterine manipulation, lesion size, lesion number, and presence of pelvic adhesion, were obtained directly from the patients’ medical records. The surgical outcomes, including pathologic findings, operating time (from skin incision to closure), estimated blood loss, transfusion, total hospital stay, recurrence, conversion rate (addition of ports), and follow-up results were compared between SILS-C and TP-C using conventional statistical methods.

### Surgical techniques

**Figure 1:**
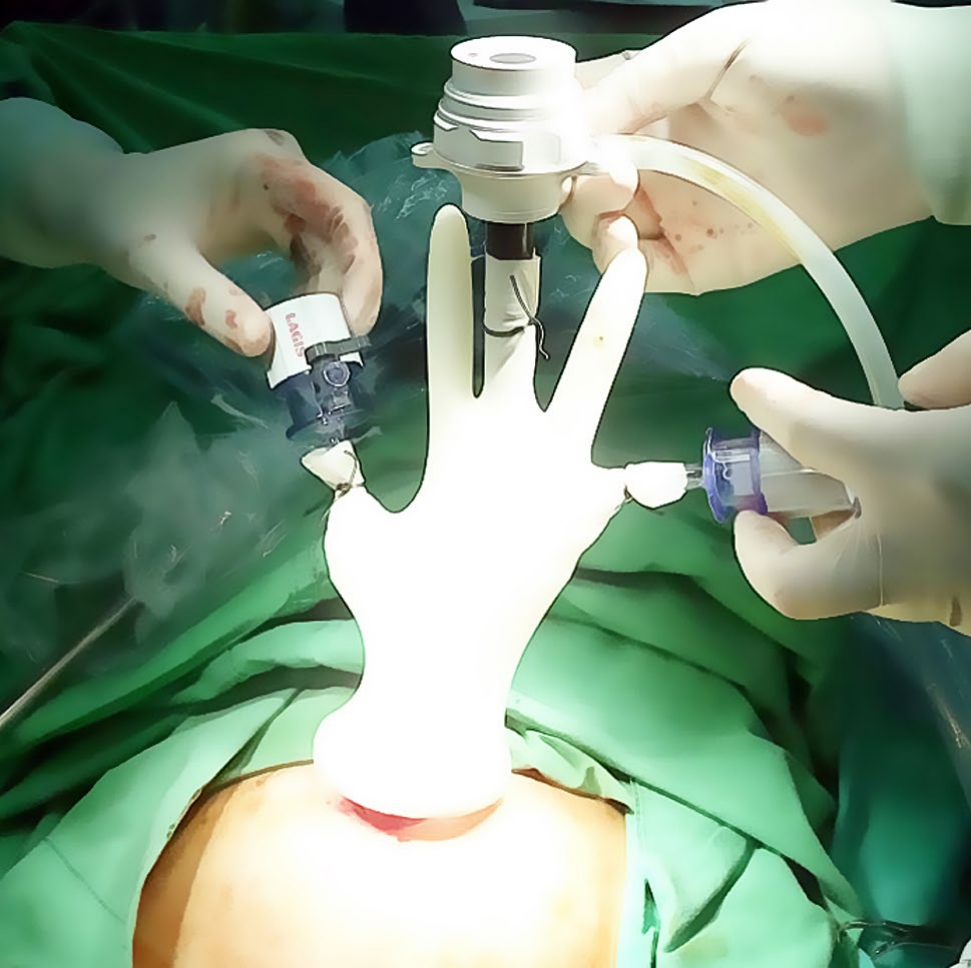
Single port laparoscopic surgery.

**Figure 2:**
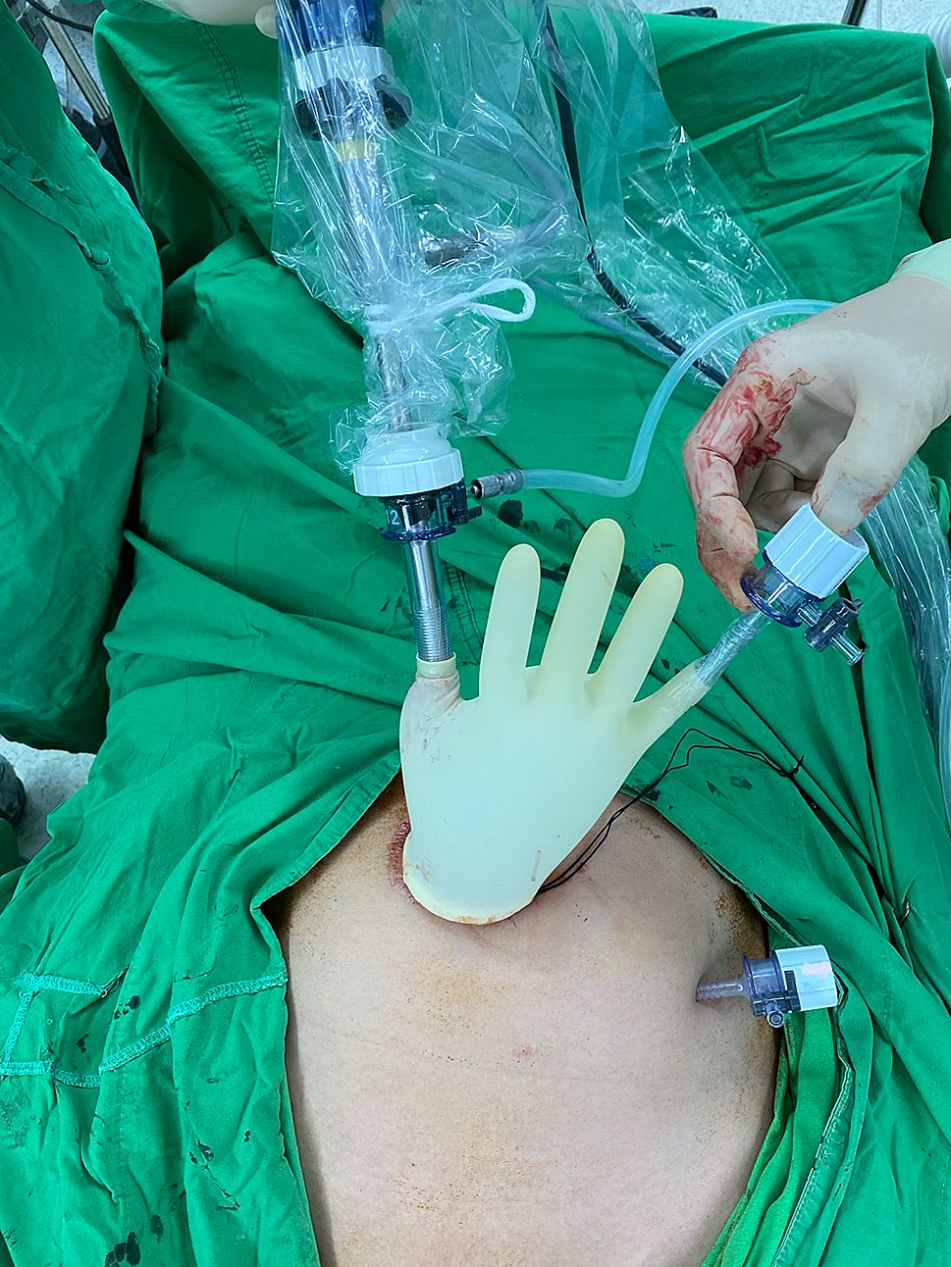
Two-port laparoscopic surgery.

After induction of general anesthesia and endotracheal intubation, patient positioning in dorsolithotomy, and placement of uterine elevator (if feasible), a 2–2.5 cm vertical skin incision was made at the umbilicus. The subcutaneous fat tissue was dissected and the peritoneum was opened. A small wound retractor (ALEXIS1, Applied Medical, Rancho Santa Margarita, CA, USA) was inserted into the wound opening transumbilically. A surgical glove with trocars inserted into three fingers was draped around the rim of the wound retractor (Fig. 1). Pneumoperitoneum was attained with the pressure set at 12–15 mm Hg. A 10-mm, 0° lens rigid laparoscope was handled by a first assistant (a senior training resident). The same method was employed for the two-port laparoscopic surgery other than the use of an additional 5-mm trocar inserted in the left lower quadrant of the abdomen (Fig. 2).

The main surgical procedures for ovarian cystectomy were the same in both surgical approaches (SILS-C and TP-C). In particular, after injecting Arginine Vasopressin (Pitressin 20 IU/ml, 1 ml diluted in Normal Saline 100 ml) into the ovarian cyst cortex as hydro-dissection, the surface was incised and stripped completely without removing any normal-appearing ovarian tissues. The stripped ovarian cyst was then removed directly (or via a modified surgical glove or Endobag) from the umbilical wound. Bleeding from the remaining ovary was controlled using bipolar electrocauterization or fibrin sealant, TISSEEL (Baxter, Westlake Village, CA). In addition, antiadhesives were placed under the patients’ request. The abdominal wall was closed via the layer-by-layer method using 1–0 Vicryl. Finally, skin closure was performed using skin adhesive.

For the adnexectomy surgery, after placement of the port(s), the ovarian ligament, fallopian tube and infundibulo-pelvic ligament of the target side were identified, dissected and cut via bipolar electrosurgical unit or Ligasure (Metronic, Minnesota, USA). The remaining procedures were the same as those for the cystectomy surgery.

For both surgical interventions the pathology types were collected and their relationships with the adhesions identified for further analysis.

### Statistical analysis

Statistical analyses were performed using SPSS Version 23.0 (SPSS Inc., Chicago, IL, USA). The collected data were analyzed by the Student’s t‐test, Fisher’s exact test, the Chi‐square test, and bivariate logistic regression. The quantitative data satisfying a normal distribution were analyzed using the Student’s t‐test (data shown as mean ± standard deviation [SD]). The qualitative data were analyzed using Fisher’s exact test and the Chi‐square test (frequency [%]). A value of *p* < 0.05 was considered as statistically significant in all tests.

## Results

A total of 643 patients were enrolled in the study. 259 patients received single incision laparoscopic surgery. The operating time was 63.83 ± 25.31 min. Blood loss was 2.38 ± 6.09 c.c. Duration of hospitalization was 3.09 ± 0.29 days. 58 patients (24.38%) needed addition of port to complete surgery. There was no complications developed.

384 patients received two-port laparoscopic surgery. In pooled analysis, the clinical characteristics of the two groups did not differ significantly; other than for the parity, which was higher in the SILS-C group than in the TP-C group (0.85 ± 1.18 vs. 0.59 ± 0.95, *p* = 0.003), and the events of anti-adhesive placement, which were higher in the TP-C group (52.9% vs. 75.52%, *p* < 0.000) (Table 1). However, the surgical outcomes between the two groups exhibited several significant differences, including a shorter operating time in the TP-C group (57.32 ± 26.38 min, OR = 0.984, CI = 0.975–0.992), a lower conversion rate (addition of port) (6.25%, OR = 0.199, CI = 0.112–0.352). Blood loss was higher (5.2 ± 19.36, OR = 1.042, CI = 1.017–1.067) (Table 2). Notably, the results were still evident after adjustment (Table 3). There was no difference in the median hospital stays of the two groups (3.09 days vs. 3.08 days, *p* = 0.711), or the recurrence rate (0.8 vs. 2%, *p* = 0.188). Moreover, none of the cases required blood transfusion or conversion to laparotomy. Endometrioma, dermoid cyst and mucinous cystadenoma were found to be the three most common types of pathology related with adhesions (Table 4).

**Table 1:** Patient characteristics by surgical approach.

	SILS-C (N = 259)	TP-C (N = 384)	*p* value
Mean age, yr (SD)	36.74 (12)	35.3 (10.95)	0.122^†^
Para (SD)	0.85 (1.18)	0.59 (0.95)	0.003^†^
BMI (SD)	22.25 (4.07)	22.46 (4.14)	0.533^†^
Previous pelvic surgery (SD)	0.32 (0.635)	0.29 (0.508)	0.560^†^
Lesion numbers (SD)	1.27 (0.46)	1.28 (0.61)	0.739^†^
Lesion size, cm (SD)	7.91 (3.76)	7.67 (3.34)	0.399^†^
Adhesion (%)	88 (33.98)	146 (38.02)	0.960^§^
Antiadhesives placement (%)	137 (52.90)	290 (75.52)	0.000^§^
Manipulation (%)	202 (77.99)	298 (77.60)	0.908^§^
Pathology* (%)	204 (78.76)	309 (80.47)	0.598^§^
**Operation methods**			
S1 versus T1 (%)	84 (52.83)	75 (47.17)	3.7^‡^
S2 versus T2 (%)	118 (31.22)	260 (68.78)	5.6^‡^
S3 versus T3 (%)	45 (69.23)	20 (30.77)	5.0^‡^
S4 versus T4 (%)	12 (29.27)	29 (70.73)	1.5^‡^

^†^Student *t* test.

^§^Chi-Square.

*The pathology with pelvic adhesion (Refer to Table 4).

^‡^The adjusted standardized residual. If the value is greater than 2, it indicates the proportion is significantly different at *p* < 0.05 level.

S1: single incision unilateral adnexectomy.

T1: two-port laparoscopic adnexectomy.

S2: single incision unilateral cystectomy.

T2: two-port laparoscopic unilateral cystectomy.

S3: single incision bilateral adnexectomy.

T3: two-port laparoscopic bilateral adnexectomy.

S4: single incision bilateral cystectomy.

T4: two-port laparoscopic bilateral cystectomy.

**Table 2:** Surgical outcomes.

	SILS-C (N = 259)	TP-C (N = 384)	*p* value
Time, min (SD)	63.83 (25.31)	57.32 (26.38)	0.002^†^
Blood loss, ml (SD)	2.38 (6.09)	5.2 (19.36)	0.008^†^
Recurrence, times (%)	2 (0.8%)	8 (2%)	0.188^†^
Hospitalization, days (SD)	3.09 (0.29)	3.08 (0.27)	0.711^†^
Conversion (%)	58 (24.38)	24 (6.25)	0.00^§^

^†^Student *t* test.

^§^Chi-Square.

**Table 3:** Outcome, adjusted.

	OR	95% CI of OR	*p* value
Time	0.984	0.975–0.992	0.000
Blood loss	1.042	1.017–1.067	0.001
Conversion	0.199	0.112–0.352	0.000

*OR* odds ratio; *CI* confidence interval.

**Table 4:** Pathology and its association with pelvic adhesion.

Type	Cases (%)	With adhesion	Without adhesion	Adjusted residual
Endometriosis	285 (44.7)	128	157	8.8
Dermoid cyst	159 (24.9)	141	18	7.6
Mucinous cyst	63 (9.8)	50	13	2.7
Serous cyst	50 (7.8)	33	17	0.4
Borderline tumor	12 (1.8)	9	3	0.8
Corpus cyst	21 (3.3)	11	10	1.1
Inclusion cyst, Brenner tumor	3 (.05)	3	0	1.3
Sex-cord stromal tumor	15 (2.4)	11	4	0.8
Malignancy	10 (1.6)	7	3	0.4
Others	19 (3)	13	6	0.4

^‡^The adjusted standardized residual. If the value is greater than 2, it indicates the proportion is significantly different at *p* < 0.05 level.

Depending on the type of adnexal surgery performed, the patients were further divided into single incision unilateral adnexectomy (S1, N = 84) and two-port laparoscopic adnexectomy (T1, N = 75); single incision unilateral cystectomy (S2, N = 118) and two-port laparoscopic unilateral cystectomy (T2, N = 260); single incision bilateral adnexectomy (S3, N = 45) and two-port laparoscopic bilateral adnexectomy (T3, N = 20); and single incision bilateral cystectomy (S4, N = 12) and two-port laparoscopic bilateral cystectomy (T4, N = 29). Except for a longer operating time in S2 than in T2 (66.12 ± 19.5 vs. 58.27 ± 23.92 min, *p* = 0.002), no statistical differences were detected in the subgroup analysis.

## Discussion

The present results suggest that single port laparoscopic surgery using conventional laparoscopic instruments takes a longer time than two-port laparoscopic surgery. Previous studies have shown (albeit, not significant in every case) shorter operating time in the CL group as a result of less complex technical requirements^1–4,17–19^. This advantage is still evident in two-port laparoscopic surgery. The conversion rates of the SILS-C and TP-C groups in the present study (22.4 and. 6.25%, respectively) are higher than those reported previously (e.g., 0 to 8% in the SILS-C group^6,20,21^). This may reflect the difference in the equipment used. For example, the present interventions were performed using a 10-mm, 0° lens, a glove port and rigid instruments, which are basically equipped and minimally required in most hospitals in Taiwan, rather than a 5-mm, 30° lens, commercialized port system and flexible instruments, which improve accessibility to the operating targets and result in less confliction between instruments.

As in some previous studies^10,21^, the blood loss was lower in the SILS-C group than the TP-C group due to a smaller total wound size. The result still reached significance compared to two-port laparoscopic surgery by only 2 ml less.

A longer operating time was observed for single port unilateral cystectomy than for two-port laparoscopic unilateral cystectomy. This finding most probably reflects the more advanced technical requirements for cystectomy than for adnexectomy. This is in line with previous report assessing the learning curve for single-port laparoscopic adnexal surgery, in which 33 cases are required for proficiency in ovarian cystectomies, while no apparent cutoff for adnexectomies^22^. No statistical significance was observed in the duration of hospitalization for the SILS-C and TP-C groups. This is most likely the result simply of hospital policy under the national insurance system in Taiwan. Besides, there is little scope to further reduce the hospitalization time in order to make clinical significance^1,6,10,11^. The recurrence rate of the SILS-C group was similar to that of the TP-C group. This may due to different follow up strategy and postoperative managements. However, the published literature contains scant information on recurrence rate, and hence it is not possible to validate the present findings.

Recently, vaginal natural orifice transluminal endoscopic surgery (NOTES) emerges with optimal cosmetic outcomes and shorter operating time^23,24^. Direct comparison between NOTES and SILS in adnexal surgery is lacking. Generally, the level of technical requirements is high. In one report, it took 36 cases for a surgeon skilled in the field of minimal invasive surgery to achieve proficiency in ovarian cystectomies^23^. Besides, this procedure may not be feasible for patients with cul-de-sac diseases^25^.

The strengths of the present study include the large sample size and the differentiation of the adnexal surgeries into different types; each of which require particular surgical techniques. The minimal equipment requirements for the laparoscopic surgery performed in the present setting made generalization of the procedure both possible and feasible. The limitations of the present study include the retrospective design, the single center experience, and possible selection bias.

In conclusion, the present results suggest that single port laparoscopic surgery using conventional laparoscopic instruments is a feasible and safe treatment for adnexal lesions. In complex setting as unilateral cystectomy or pelvic adhesions, two-port access may be considered. Further randomized controlled studies are suggested for confirmation purposes.
