# Author Correction: Single incision laparoscopic surgery using conventional laparoscopic instruments versus two-port laparoscopic surgery for adnexal lesions

**DOI:** 10.1038/s41598-021-98468-w

**Published:** 2021-09-14

**Authors:** Kuan‑Ju Huang, Kuan‑Ting Lin, Chin‑Jui Wu, Ying‑Xuan Li, Wen‑Chun Chang, Bor‑Ching Sheu

**Affiliations:** 1 Department of Obstetrics and Gynecology, National Taiwan University Hospital, National Taiwan University College of Medicine, 7 Chung‑Shan South Road, Taipei, Taiwan; 2 National Taiwan University Hospital Hsin-Chu Branch, Hsichu City, Taiwan

Correction to: *Scientific Reports* 10.1038/s41598-021-82204-5, published online 18 February 2021

Figure 2 in the original version of this Article is a duplication of Figure 2 in the article “Huang, KJ., Li, YX., Sheu, BC. et al*.* Two-port access for laparoscopic surgery for endometrial cancer using conventional laparoscopic instruments. *Sci Rep* **11,** 615 (2021). 10.1038/s41598-020-79886-8.” Both of these studies describe the same surgical technique for laparoscopic surgery, for which Figure 2 is a representative image.

